# Intraocular pressure correlation to progressive retinal nerve fiber layer loss in primary open angle glaucoma as measured by standard and modified goldmann applanation tonometers

**DOI:** 10.1186/s12886-025-04060-5

**Published:** 2025-04-30

**Authors:** Sean McCafferty, Manjool Shah, Anupam Laul, Khin Kilgore

**Affiliations:** 1 Associate Clinical Professor, University of Arizona COM, Department of Ophthalmology, Arizona Eye Consultants, 6422 E. Speedway Blvd., Tucson, AZ 85710 USA; 2 Associate Professor, University of Michigan, Kellogg Eye Center, 1000 Wall St, Ann Arbor, MI 48105 USA; 3 Associate Clinical Professor, SUNY College of Optometry, 33 W 42 ST., NEW YORK, NY 10036 USA; 4 Arizona Eye Consultants, 6422 E. Speedway Blvd., Tucson, AZ 85710 USA

**Keywords:** Tonometry, OCT, Retinal nerve fiber layer, Corneal biomechanics, Glaucomatous optic neuropathy, Glaucoma, Corneal hysteresis

## Abstract

**Purpose:**

Characterize the relationship between intraocular pressure (IOP) as measured by standard and modified Goldmann prisms and the progressive loss of retinal nerve fiber layer (RNFL) in a cohort of glaucoma patients.

**Design:**

Retrospective cross-sectional cohort data analysis.

**Participants:**

The study included all patients from a database of 1927 eyes, 966 patients with same visit sequential *standard* and *modified* Goldmann IOP measurements. From the database, 148 eyes, 75 patients met the inclusion criteria of a diagnosis of primary open angle glaucoma (POAG) with at least 5 sequential quality optical coherence tomographer (OCT) measurements.

**Methods:**

Sequential OCT images were obtained with the spectral domain Zeiss OCT5000. Participants were all diagnosed with POAG by untreated IOP ≥ 22, disk changes, and visual field (HVF) loss consistent with glaucomatous optic neuropathy (GON). Included were 575 Goldmann IOP measurements with *standard* and *modified* prisms affixed to the Goldmann applanation tonometer (GAT) armature. A modified prism includes a corneal conforming applanation surface minimizing the cornea’s contribution to the IOP measurement. The study included a total of 940 OCT visits with an average of 6.3 visits per eye over an average of 4.9 years. Retinal nerve fiber layer (RNFL) loss rate was calculated by serial linear fit of average RNFL thickness measurements. Demographics as well as central corneal thickness (CCT) and corneal hysteresis (CH) data were also collected.

**Outcome measures:**

Pearson correlation coefficients and random coefficient models were used to evaluate the relationship between mean *standard* and *modified* IOP measurements and RNFL thickness measurements over time in POAG subjects. Secondary outcomes of CCT and CH correlation to RNFL were similarly analyzed.

**Results:**

For all 148 POAG eyes, the overall rate of RNFL loss for an average *standard* GAT IOP of 17.9 mmHg was 1.08 µm per year (*p* = 0.002). Each 1-mmHg increase in *standard* GAT IOP was associated with an additional RNFL loss of 0.047 µm per year (*r* = 0.153, *p* = 0.06). Each 1-mmHg increase in *modified* GAT IOP was associated with an additional RNFL loss of 0.084 µm per year (*r* = 0.289, *p* = 0.0005). A *modified* prism IOP measurement ≥ 22 mmHg indicates a 2.57 times greater probability of significant RNFL loss than a *standard* prism IOP measurement ≥ 22 mmHg, *p* < 0.0001.

**Conclusions:**

Higher levels of GAT IOP during follow-up were related to higher rates of progressive RNFL loss detected by optic nerve OCT in treated POAG. A *modified* GAT prism surface demonstrates a significantly increased sensitivity, reliability and differentiation to progressive RNFL loss when compared to a standard GAT prism measured IOP.

**Précis:**

A modified applanation surface prism with a corneal conforming shape used on a Goldmann tonometer appears to be a more sensitive and reliable indicator of progressive glaucomatous optic neuropathy as measured by retinal nerve fiber layer changes.

## Background

The primary risk factor for progression of glaucomatous optic neuropathy (GON) has been identified as intraocular pressure (IOP) in several clinical trials [1–4, 6]. The Early Manifest Glaucoma Trial (EMGT) demonstrated each additional 1-mmHg of increased IOP was correlated with an average of 10% increased visual field loss [5]. At the opposite end of the of the POAG spectrum, the Advanced Glaucoma Intervention Study (AGIS) related less visual field loss with lower IOP in those with advanced POAG [4]. Even among those in a pre-glaucomatous state, the Ocular Hypertension Treatment Study (OHTS) showed that pharmacological lowering of intraocular pressure (IOP) was associated with a 54% reduced relative risk of developing POAG [7].

Although automated perimetry has been a baseline standard for the detection of GON progression, other imaging modalities measuring structural changes in the optic nerve have been shown to precede visual field loss [2, 3]. Changes detected by RNFL imaging are shown to often be the earliest indication of GON prior to associated visual field changes [8, 9]. RNFL imaging devices quantitatively measure the nerve fiber thickness and are a mainstay in diagnosis and treatment of glaucoma. Methods for evaluating the RNFL include spectral domain optical coherence tomography (SD-OCT) [10–14]. One clinical SD-OCT device commercially available is the Cirrus Zeiss OCT5000 (Carl Zeiss Meditec, Inc., Dublin, CA) which provides an objective and reproducible evaluation of the RNFL.

We know from, Medieros et al., that there is a positive relationship between Goldmann IOP and progressive RNFL loss in POAG patients by longitudinally measuring RNFL [15]. Eyes with POAG and glaucoma suspects progression were found to have an overall RNFL loss of 0.95 µm/year and 0.13 µm/year per increase of 1 mmHg. The Goldmann applanation tonometer (GAT) was used as the IOP reference measurement in all the aforementioned studies.

A *modified* Goldmann prism or correcting applanation tonometry surface (CATS) prism utilizes a centrally concave and peripherally convex surface, which partially matches corneal curvature. The altered prism is affixed to the Goldmann tonometer and uses the same measurement protocol without recalibration [16–20]. The prism was designed to minimize the IOP measurement errors found with the *standard* flat GAT prism [18]. Clinical studies using the *modified* prism have demonstrated that its IOP measurements are less influenced by variations in CCT, corneal hysteresis (CH), and tear film compared to the *standard* prism [16–20]. The purpose of this study is to examine the differential correlation in a cohort of glaucoma patients by measuring IOP with a *standard* flat surfaced Goldmann prism and a *modified* corneal conforming Goldmann prism to longitudinal RNFL changes measured by SD-OCT.

## Methods

The study design was as a cross-sectional cohort study. Participants were included in a retrospective, longitudinal analysis to examine the correlation of optic nerve structural changes to IOP measured by Goldmann applanation tonometry using a *standard* and *modified* prism. Participants in this study were cross-sectionally provided without personal identifiable data according to a pre-established protocol between 1–1–20 and 2–15–23 at three participating centers within the Arizona Eye Consultants (Tucson, AZ) practice. These records included SD-OCT5000 measurement data with a maximum range of 1–1–14 to 2–15–23. All participants were undergoing routine follow-up visits for POAG diagnosed by standard diagnostic criteria (listed below) with required imaging and field tests. Deidentified participant data was entered into an electronic medical record database for collating and retrieval. All protocols and the methods described adhered to the tenets of the Declaration of Helsinki.

Subjects were undergoing comprehensive ophthalmologic examinations at each visit. Examination data included deidentified medical history and medication review, slit-lamp biomicroscopy, best-corrected visual acuity, IOP measurement using calibrated Goldmann applanation tonometry (GAT), dilated fundoscopic examination, gonioscopy, optic disc photography, and automated perimetry using 24–2 Full-threshold. Only subjects with gonioscopically demonstrated open angles were included. Subject exclusion criteria included less than 20/80 best-corrected visual acuity, more than ± 6.0 diopters refractive error, or cylinder correction greater than 3.0 diopters. Participant’s data was excluded if it was found to have any other systemic or ocular disease that could affect the optic nerve or visual field testing.

The study data included patients diagnosed with POAG and paired IOP measurements completed with the *standard* and *modified* Goldmann prisms. Ensuring the strictest definition, data was included if eyes were classified as having glaucoma having 2 repeatable abnormal visual field tests, indicated by a pattern standard deviation outside of the 95% normal confidence limits. Specifically, eyes were classified as POAG if they included a history of *standard* GAT measured IOP (> 21 mmHg) without signs of secondary open angle glaucoma. Bilateral eligible eyes from a given patient’s data were included in the analysis where applicable. Statistical procedures were used to take into account the correlation between measurements within the same patient (see below).

POAG subjects with a minimum 5 follow-up visits in which high quality SD-OCT measurements were obtained and at least 2 same visit sequential Goldmann IOP measurements using both the *standard* and *modified* prisms were required for inclusion in this study. Quality OCT measurement excluded those with decentration, artifacts or optical image drop-out near the 3.2 mm measurement circumference.

Intraocular pressure measurements were completed on a calibrated Goldmann applanation tonometer BM- 900 (Haag-Streit, Bern, Switzerland). Measurement techniques were in accordance with the tonometer instructions for use (IFU) and ANSI Z80.10–2014/18- Annex A- A.3 Protocol for using the reference tonometer [21].

Sequential OCT images were obtained with the spectral domain Zeiss OCT5000 (Carl Zeiss Meditec, Inc., Dublin, CA). Participants had been diagnosed with POAG by untreated IOP > 21, disk changes, and visual field (HVF) loss consistent with glaucomatous optic neuropathy (GON). Retinal nerve fiber layer (RNFL) loss rate was calculated by serial linear fit of global average RNFL thickness data measurements. The RNFL thickness measurements were obtained on a 3.2-mm diameter calculation circle around the optic nerve head. In addition to demographics data, central corneal thickness (CCT) was obtained using a Zeiss OCT5000 (Carl Zeiss Meditec, Inc., Dublin, CA) and corneal hysteresis (CH) was obtained using the Ocular Response Analyzer (ORA) (Reichert Technologies, Buffalo, NY).

Statistical Analysis using random coefficient models were used to evaluate the relationship between IOP and RNFL thickness measurements over time. Previous studies have used these general linear mixed effects (GLME) models with randomized slopes and intercepts to investigate the correlation of RNFL to multiple variables in glaucoma [22, 23]. The model was constructed evaluating relationships between IOP and time dependent OCT RNFL thickness measurements. Linear regression SD-OCT RNFL thickness measurements were designated as the dependent variable and the time varying predictor was IOP.

Almost all patients had a left and right eye included so the effects of patient laterality was included in the GLME. It was modeled as a random effect and intercept alongside fixed effect coefficients. Random intercept inclusion allowed for baseline RNFL variation and randomized slope coefficients allow for progressive RNFL loss variations among eyes and patients. Random effects models were estimated in the R programming language, R-version 4.3.3, using maximum likelihood and the function lme() from the nlme package (R Project for Statisical Computing).

Each model included patient as a random intercept effect. Fixed effect covariates included eye, sex, race, age, and average Mod-Std IOP difference (M-S.avg). Because approximately 20% of patients had missing values for corneal hysteresis (CH), estimation occurred with and without the CH variable. The inclusion of random intercepts allows for the variation in baseline RNFL, whereas the random slopes allow for the variation in the rate of progressive RNFL loss among eyes and patients.

The general basis form of the models for an IOP measurement was as follows:

$$IOP=\left({\beta \left(0\right)}^{GAT}+{I}^{CATS}*{\beta \left(0\right)}^{\Delta }+\beta {\left(0\right)}^{cct}*CCT+\beta {\left(0\right)}^{CH}*CH+\beta {\left(0\right)}^{age}*Age\right)+\left({\beta \left(1\right)}^{GAT}+{I}^{CATS}*{\beta \left(1\right)}^{\Delta }+\beta {\left(1\right)}^{cct}*CCT+\beta {\left(1\right)}^{CH}*CH+\beta {\left(1\right)}^{age}*Age\right)*RNFL Loss+(1+RNFL Loss | Eye, Race, Sex)$$

Subsequent models were built with and without corneal hysteresis (CH) and separately Mod and Std IOP as well as same day Mod-Std differential IOP.

## Results

The cross-sectional study included deidentified data from all patients from the EMR database examined between the dates of 1–1–20 and 2–15–23. The collection data included 1927 eyes from 966 patients with same visit sequential *standard* and *modified* Goldmann IOP measurements. From the IOP database pool, the full analysis dataset contained 148 eyes from 75 patients met the inclusion criteria of a diagnosis of primary open angle glaucoma (POAG) with at least 5 sequential quality optical coherence tomographer (OCT) measurements. Annualized RNFL was estimated for each patient eye via linear regression on RNFL readings, with time as the predictor variable. RNFL regression ranged from − 6.27 to 2.32 microns, with mean − 1.08 and standard deviation 1.30.

The full analysis data set Included 575 Goldmann IOP measurements with *standard* and *modified* prisms affixed to the calibrated Goldmann applanation tonometer (GAT) armature. The study included a total of 940 OCT visits with an average of 6.3 ± 1.4 visits per eye over an average of 4.9 ± 1.5 years.

Average values of the RNFL thickness throughout the evaluation period was 74 ± 13 µm. Paired *modified* minus *standard* IOP measurements averaged 3.8 ± 1.6 mmHg per patient over the collection time interval.

There were 9 predictor variables, described below.

1. Mod IOP avg: Patient eye average intraocular pressure from the newer *modified* prism, in mm Hg. Ranges from 12.00 to 42.50 mmHg, with mean 19.97 ± 4.37 mmHg.
2. Std IOP avg: Patient eye average intraocular pressure from the *standard* prism, in mm Hg. Ranges from 9.00 to 39.50 mmHg, with mean 17.95 ± 4.23 mmHg.
3. Mod. IOP minus Std. IOP avg: Patient eye average difference (Mod. – Std.), only computed when both measurements are taken during the same visit. Ranges from − 2 to 6 mmHg, with mean 1.92 ± 1.73 mmHg standard deviation. If a patient had only one pressure measurement during a visit, that measurement is included in the Mod. or Std. IOP average, but was not used in this differential (Mod.-Std.) IOP variable. Therefore, this variable is not identical to (Mod. IOP avg – Std. IOP avg).
4. CH avg: Patient eye average corneal hysteresis. Ranges from 3.70 to 13.55 mmHg, with mean 9.03 ± 1.66 mmHg standard deviation. Of the 75 patients, 14 did not have measured values for this variable.
5. Eye: Indicator variable for OD (right) or OS (left) eye.
6. Age: Highest age in years recorded for each patient on any visit. Ranges from 13 to 92, with mean 73.16 ± 11.20 standard deviation.
7. Sex: Reported sex of the patient. 47 patients were female and 28 were male.
8. Race: Reported race/ethnicity of the patient. Caucasian was selected for 50 patients (67%), Non-white/Hispanic for 15 (20%), African-American for 8 (11%), and Asian for 2 (2.7%).
9. CCT: Measured central corneal thickness. Mean 542 ± 49 µm standard deviation.

### Correlations and regressions without covariates

**Fig. 1:**
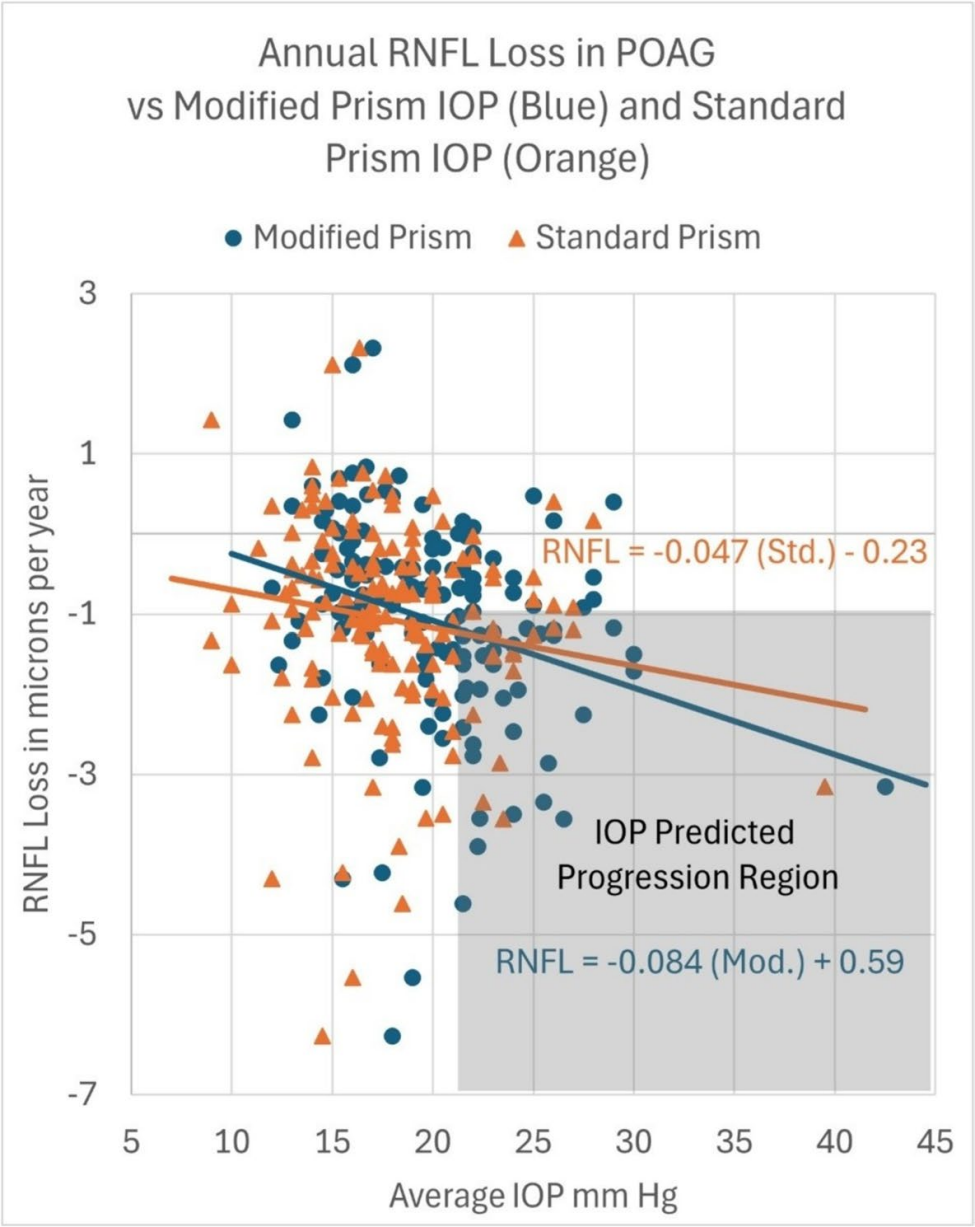
**-** RNFL loss in POAG (annualized) verses average IOP measured by a *standard* and *modified* Goldmann prism

For all 148 POAG eyes, the overall rate of RNFL loss for an average *standard* GAT IOP of 17.9 mmHg (19.9 mmHg by *modified* prism) was 1.08 ± 1.30 µm per year (*p* = 0.0005). Demonstrated in Fig. 1, Each 1-mmHg increase in *standard* GAT IOP was associated with an additional RNFL loss of 0.047 µm per year (*r* = 0.153, *p* = 0.06). Also demonstrated in Fig. 1, Each 1-mmHg increase in *modified* GAT IOP was associated with an additional RNFL loss of 0.084 µm per year (*r* = 0.289, *p* = 0.0005).

The graph in Fig. 1 shows RNFL loss per year as the vertical variable, with Mod and Std average IOP readings on the horizontal axis. Orange triangles represent Std IOP readings, while blue circles represent Mod IOP readings. Univariate regression lines are included. Higher measurements of *modified* and *standard* IOP are associated with more negative slopes of RNFL loss per year. The modified prism IOP slope is statistically significant at *p* = 0.0005 and the standard prism IOP slope approaches significance at *p* = 0.06 (Table 1).

**Table 1:** *Modified* and *standard* prism IOP regression analysis output summary

ANOVA					
*Modified Prism*	*Deg of freedom*	*Sum of squares*	*Mean square*	*F-statistic*	*Significance F-statistic*
Regression	1	19.69	19.69	12.56	0.00052
Residual	146	228.84	1.57		
Total	147	248.539			
*Standard Prism*	*Deg of freedom*	*Sum of Squares*	*Mean square*	*F-statistic*	*Significance F-statistic*
Regression	1	5.84	5.84	3.51	0.062
Residual	146	242.70	1.66		
Total	147	248.53			

To examine the differences between *modified* and *standard* slopes, an interaction term was created. The interaction term represents a difference in slope between the two lines. The interaction coefficient value was not statistically significantly different from zero (*p* = 0.299). Although the *Modified* prism slope is steeper at − 0.084 µm/yr/mmHg compared to the *standard* slope of − 0.047 µm/yr/mmHg, claiming that the slopes differ significantly in unpaired measurements is not supported, as the 95% confidence interval for the *modified* slope of (− 1.474, − 0.419) contains the *standard* slope.

**Fig. 2:**
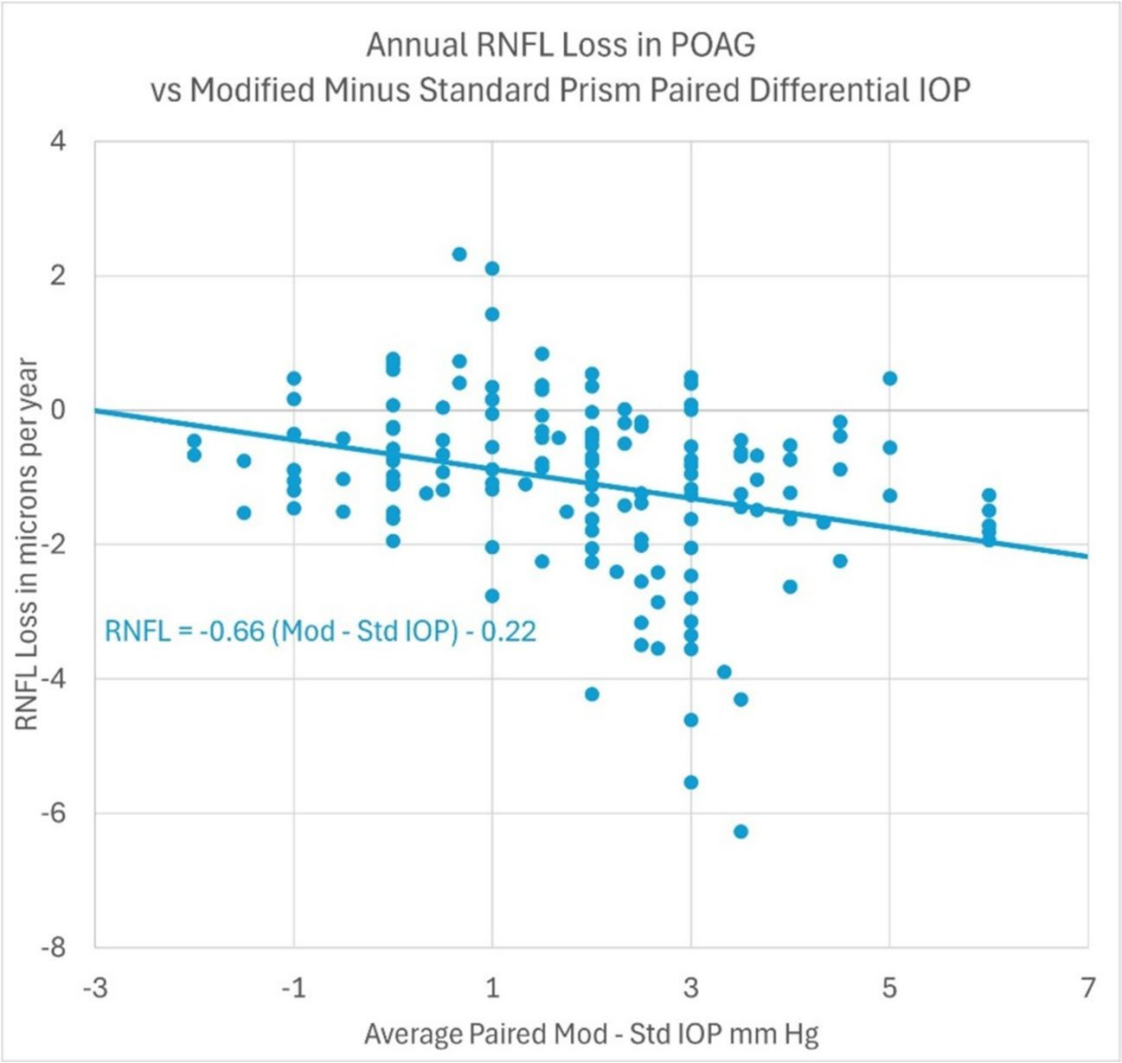
**-** RNFL loss in POAG (annualized) verses average paired differential IOP *modified* minus *standard* Goldmann prism measurements

There is a possibility of insufficient study power in unrelated *standard* and *modified* IOP measurement slope differences correlated to RNFL loss due to time and operator variations in IOP measurement. To account for this, a same-time IOP measurement (paired) differential between *modified* and *standard* prisms was examined to directly interpret the difference in average IOP as it correlates to RNFL loss in POAG. IOP which was measured by *standard* or *modified* prisms at separate times were excluded. Figure 2 illustrates each 1 mmHg difference between *modified* and *standard* prism IOP measurements was significantly correlated to RNFL loss at 0.66 µm per year (*r* = 0.289, *p* = 0.0004) (Table 2).

**Table 2:** *Modified* minus *standard* prism differential IOP regression analysis output summary

ANOVA					
*Mod-Std IOP*	*Deg of freedom*	*Sum of squares*	*Mean square*	*F-statistic*	*Significance F-statistic*
Regression	1	20.80312	20.80312	13.3371104	0.000362
Residual	146	227.7297	1.559792		
Total	147	248.5328			

CCT did not correlate to RNFL loss in treated POAG (*p* = 0.51). Without including the known CH covariate of IOP, each 1-mmHg decrease in CH was associated with an additional RNFL loss of 0.244 µm per year (*p* = 0.0001).

In the table of Pearson correlations between continuous variables, Mod. IOP average and Std. IOP average have a strong correlation of + 0.915 between them. Mod average has a greater correlation strength with the response, magnitude 0.281 against 0.153 for Std (Table 3).

**Table 3:** Pearson correlation strength between continuous variables

	*RNFL Loss µm/yr*	*Mod.IOP.avg. mmHg*	*Std.IOP.avg. mmHg*	*Mod.-Std.avg. mmHg*	*CCT µm*	*CH.avg. mmHg*	*Age yrs*
RNFL Loss/yr	1						
Mod.IOP.avg	− 0.281	1					
Std.IOP.avg	− 0.153	0.915	1				
Mod.-Std.avg	− 0.289	0.317	− 0.030	1			
CCT	0.003	− 0.007	0.013	− 0.021	1		
CH.avg	0.341	− 0.350	− 0.254	− 0.257	− 0.011	1	
age	− 0.133	0.011	− 0.042	0.105	− 0.010	− 0.091	1

Ocular hypertension can be defined as an IOP ≥ 22 mmHg measured by either a modified or standard Goldmann prism. Furthermore, significant RNFL loss progression can be defined as ≥ 1.0 µm/yr. The lower right quadrant (labeled IOP predicted progression region) in Fig. 1 inclusive of the lines delineated by x = 22 mmHg vertically and y = 1.0 µm/yr. horizontally can be used to can be used to calculate the relative risk of RNFL progression ≥ 1.0 µm/yr. This analysis indicates an IOP measurement ≥ 22 mmHg with the *modified* prism is 2.57 times more likely to demonstrate significant RNFL loss than a *standard* prism at the same IOP, *p* < 0.0001.

### Multivariate models with covariates

Building the multivariate models almost all patients have a left and right eye, so the effects of the patient were included. Patient effects were modeled as a random effect alongside fixed effect coefficients. Random effects models were estimated in the R programming language, version 4.3.3, using maximum likelihood and the function lme() from the nlme package.

Each model included patient as a random intercept effect. Fixed effect covariates included eye, sex, race, CCT, age, and average Mod-Std difference (Mod-Std.avg). Because approximately 20% of patients had missing values for corneal hysteresis (CH), estimation occurred with and without the CH variable. There are a total of 6 models, 2 with CH and 4 without CH. Each set includes models with neither Mod IOP nor Std IOP, Mod IOP only, Std IOP only, both Mod IOP and Std IOP.

### Model summary results from GLME

Summaries of fixed effect results from each of the 6 GLME models. The “ ~ ” symbol separates the response from predictor variables. Output includes each coefficient, standard error, *t*-value, and *P*-value. The Models included Standard IOP alone, Modified IOP alone, Standard and Modified IOP together, Differential Mod.- Std. IOP, and corneal hysteresis with either Standard or Modified IOP.

Modeled covariates tend to have consistency across models, as described below.

- Higher values of *modified* prism IOP are always associated with more negative regression slopes for RNFL loss. This aligns with the univariate result. In both models with and without CH, the coefficient is statistically significant at the 5% level. (Tables 5, 6 and 9)
- Higher values of *standard* prism IOP are always associated with more negative regression slopes for RNFL loss. This aligns with the univariate result. In models without CH, the coefficient approaches statistically significant at the 5% level.(*p* = 0.027,0.072) (Tables 4 and 6). In the model with CH, it does not reach significance (*p* = 0.113) (Table 8)
- The Mod. – Std. differential IOP (Mod-Std) is significantly associated with a more negative slope of RNFL loss, *p* = 0.0007 (Table 7). On the other hand, in the models including both Std IOP and Mod IOP, this differential IOP (Mod.-Std) variable has an insignificant impact on RNFL loss slope. This change is not surprising, as direct measurements are often more informative than a computed difference between direct measurements.(Table 6)
- Age, Race, CCT and Eye never have a significant impact on the slope of RNFL loss. (Tables 4, 5, 6, 7, 8 and 9)
- Being male has an impact of around − 0.40 microns per year, compared to being female. This coefficient is never statistically significant at the 5% level, but the P-values are between 0.073 and 0.085 in all six models. (Tables 4, 5, 6, 7, 8 and 9)
- Decreased corneal hysteresis (CH) always has a significant negative impact on slope of RNFL loss, with an increase between 0.14 and 0.17 microns of RNFL loss for each 1 mmHg decrease in CH level. P-values range from 0.028 to 0.035. (Tables 8 and 9)

**Table 4:** Standard Prism only: Fixed effects: regress.yr ~ Std.IOP.avg + age + sex + race + Eye + CCT

Column1	Reg. Coeff	Std. Error	Deg of Freedom	t-value	p-value
Intercept	1.4779	0.9296	70	1.5898	0.1164
Std.IOP.avg mmHg	− 0.0484	0.0265	70	− 1.8237	0.0725
Age yrs	− 0.1337	0.0108	69	− 1.2378	0.2201
Sex Male	− 0.4277	0.2454	69	− 1.7431	0.0858
Race African	0.0624	0.4196	69	0.1487	0.8822
Race Asian	0.0003	0.7091	69	0.0005	0.9996
Race Hispanic	− 0.2469	0.29422	69	− 0.8392	0.4042
Eye OS	− 0.1297	0.1774	70	− 0.7309	0.4672
CCT µm	− 0.0305	0.1633	70	− 0.6377	0.5002

**Table 5:** Modified Prism only: Fixed effects: regress.yr ~ Mod.IOP.avg + age + sex + race + Eye + CCT

Column1	Reg. Coeff	Std. Error	Deg of Freedom	t-value	p-value
Intercept	1.7659	0.9283	70	1.9023	0.0612
Mod.IOP.avg mmHg	− 0.0652	0.0269	70	− 2.4247	0.0179
Age yrs	− 0.0128	0.0107	69	− 1.2006	0.234
Sex Male	− 0.4341	0.2428	69	− 1.7878	0.0782
Race African	0.1338	0.4153	69	0.3222	0.7482
Race Asian	0.005	0.7014	69	0.0071	0.9943
Race Hispanic	− 0.2893	0.2921	69	− 0.9909	0.3252
Eye OS	− 0.127	0.1759	70	− 0.7222	0.4726
CCT µm	− 0.0295	0.1661	70	− 0.6345	0.5085

**Table 6:** Standard and modified prism together and paired differential IOP, Mod.IOP-Std.IOP: Fixed effects: regress.yr ~ Std.IOP.avg + Mod.IOP.avg + age + sex + race + Eye + CCT + Mod.IOP-Std.IOP

Column1	Reg. Coeff	Std. Error	Deg of Freedom	t-value	p-value
Intercept	1.821	0.9181	69	1.983523	0.0513
Mod.IOP.avg mmHg	− 0.3162	0.1143	69	− 2.7661	0.0073
Std.IOP.avg mmHg	0.2525	0.1118	69	2.2577	0.0271
Age yrs	− 0.0121	0.0106	69	− 1.1462	0.2557
Sex Male	− 0.4344	0.2402	69	− 1.8087	0.0748
Race African	0.1051	0.4109	69	0.2557	0.7989
Race Asian	− 0.0619	0.6944	69	− 0.0892	0.9291
Race Hispanic	− 0.4304	0.2955	69	− 1.4565	0.1498
Eye OS	− 0.1597	0.1734	69	− 0.9215	0.3601
CCT µm	− 0.0093	0.0599	69	− 0.5667	0.4045
Mod.IOP-Std.IOP mmHg	0.04596	0.1176	69	0.3883	0.6989

**Table 7:** Paired differential IOP, Mod.IOP-Std.IOP: Fixed effects: regress.yr ~ age + sex + race + Eye + CCT + Mod.IOP-Std.IOP

Column1	Reg. Coeff	Std. Error	Deg of Freedom	t-value	p-value
Intercept	0.7054	0.8424	71	0.8373	0.4052
Age yrs	− 0.0143	0.0109	69	− 1.3051	0.1962
Sex Male	− 0.4097	0.2496	69	− 1.6411	0.1053
Race African	0.1599	0.4088	69	0.3913	0.6968
Race Asian	− 0.0618	0.7214	69	0.0856	0.9321
Race Hispanic	− 0.2303	0.2995	69	− 0.7689	0.4445
Eye OS	− 0.1614	0.1763	71	− 0.9157	0.3629
CCT µm	− 0.0039	0.0404	71	− 0.5034	0.3885
Mod.IOP-Std.IOP mmHg	− 0.2281	0.1642	71	− 3.5527	0.0007

**Table 8:** Average Corneal Hysteresis with Avg. Standard Prism IOP: Fixed effects: regress.yr ~ Std.IOP.avg + CH.avg + age + sex + race + Eye + CCT

Column1	Reg. Coeff	Std. Error	Deg of Freedom	t-value	p-value
Intercept	− 0.3023	1.1103	56	− 0.2722	0.7864
Std.IOP.avg mmHg	− 0.0401	0.0249	56	− 1.6115	0.1127
CH.avg mmHg	0.1469	0.0653	56	2.2511	0.0283
Age yrs	− 0.0092	0.0092	55	− 1.0159	0.3141
Sex Male	− 0.4301	0.2185	55	− 1.9673	0.0542
Race African	0.0766	0.3677	55	0.1948	0.8462
Race Asian	− 0.3503	0.8103	55	− 0.4322	0.6672
Race Hispanic	0.1944	0.2906	55	0.6689	0.5064
Eye OS	− 0.2112	0.1872	55	− 1.1316	0.2626
CCT µm	− 0.0083	0.1009	56	− 0.4304	0.6465

**Table 9:** Average Corneal Hysteresis with Avg. Modified Prism IOP: Fixed effects: regress.yr ~ Mod.IOP.avg + CH.avg + age + sex + race + Eye + CCT

Column1	Reg. Coeff	Std. Error	Deg of Freedom	t-value	p-value
Intercept	− 0.0833	1.1194	56	− 0.0744	0.9409
Mod.IOP.avg mmHg	− 0.0489	0.0253	56	− 1.9936	0.0482
CH.avg mmHg	0.1411	0.0651	56	2.1663	0.0346
Age yrs	− 0.0092	0.0092	55	− 1.0021	0.3207
Sex Male	− 0.4312	0.2175	55	− 1.9816	0.0525
Race African	0.1011	0.3656	55	0.2766	0.7831
Race Asian	− 0.3717	0.8065	55	− 0.4608	0.6467
Race Hispanic	0.1763	0.2895	55	0.6091	0.545
Eye OS	− 0.2111	0.1862	55	− 1.1339	0.2616
CCT µm	− 0.0088	0.1599	56	− 0.4139	0.6805

## Discussion

The Pearson correlation coefficient indicates an increased reliability and predictive value to progressive RNFL loss in treated POAG by the *modified* prism IOP measurement compared to the *standard* prism IOP measurement. The steeper slope of 0.084 µm/yr/mmHg per year with the *modified* prism compared to 0.047 µm/yr per year in *standard* prism provides a greater sensitivity and differentiation in measurement predicting RNFL loss. Furthermore, each 1 mmHg difference between *modified* and *standard* prism IOP measurements was associated with RNFL loss at 0.66 µm per year. The increased *modified* prism IOP reliability is supported by the multivariate analyses with *standard* prism correlations similar to prior studies [15].

In both the univariate and multivariate analysis, the *modified* prism IOP measurement demonstrated more usefulness than the *standard* prism IOP measurement when predicting the rate of RNFL loss. There are several indications: *Modified* prism IOP has a stronger correlation magnitude and statistical significance in average *modified* prism IOP and paired differential (*modified-standard*) IOP. In the multivariate random effects models, *modified* prism IOP coefficients show statistical significance more often both individually and in paired differential IOP measurements. When both *standard* prism IOP and *modified* prism IOP variables are entered, the *modified* prism IOP maintains the proper direction in the counter-balance collinear effect, indicating greater predictive strength.

Using standard benchmarks of ocular hypertension with an IOP ≥ 22 mmHg and a significant progressive RNFL loss (average) > 1.0 µm/yr., a prognostic relative risk can be calculated between the *modified* and *standard* prism IOP measurements. A *modified* prism IOP measurement ≥ 22 mmHg indicates a 2.57 times greater probability of significant RNFL loss compared to a *standard* prism IOP measurement ≥ 22 mmHg in treated POAG. Healthy subjects estimated an age-related loss of 0.08 µm/year in average RNFL thickness [24]. The average rate of RNFL loss in the present study of progressing POAG eyes (> 1.0 µm per year, or > 1.5% per year) was 2.14 µm per year compared with only 0.27 µm per year (< 0.5% per year) in non-progressing POAG (*P* = 0.01). This large difference in *modified* and *standard* prism measured IOP as an indicator of POAG progression (2.57 times) could support a hypothesis of decreased incidence in normal tension glaucoma (NTG) as measured by the *modified* prism for future examination.

There is a strong association in the multivariate analysis between decreased corneal hysteresis (CH) and progressive RNFL loss even when IOP is included as a covariate measured by *standard* or *modified* prisms. These findings indicate CH may have a separate effect on glaucoma progression than IOP as found in previous studies examining CH and RNFL loss [25, 26]. Zhang, et al. found that each 1 mm Hg decrease in CH was associated with a 0.13 μm/year increase in RNFL loss which is almost identical to our 0.14 to 0.15 μm/year increase in RNFL loss in the present study’s multivariate analysis [26].

It is possible that the increased RNFL loss with lower *standard* IOP measurements compared to *modified* prism IOP measurements is associated with a significant clinician reliance on IOP in assessing early signs of POAG progression initiating more aggressive treatment which is independent of RNFL or HVF changes. Multiple studies support a decreased IOP error with the *modified* corneal conforming surface [17–20]. No significant *standard* verses *modified* prism IOP bias has been demonstrated in healthy, disease-free patients [16, 17].

The multivariate analysis with the limited sample size indicates no effect on treated POAG progression indicated by RNFL loss associated with CCT. The study analysis found no association of RNFL loss with age, eye, and race in treated POAG. Although the association between male gender and increased RNFL loss was not statistically significant at *p* = 0.054 to *p* = 0.105, it may have been with a larger sample size, which has been noted in other studies [27]. Although the *modified* prism IOP has been shown to correct for CCT error, it also has been shown to simultaneously correct for corneal rigidity, curvature, and tear film possibly accounting for its more significant association with glaucoma progression than the *standard* prism IOP [16–20].

Quadrant specific TSNIT and bifurcated high and low progression rates analyses could be conducted with a larger study. Rates of OCT RNFL loss were higher for the inferior and superior sectors around the optic nerve in an associated study and in agreement with neuroretinal rim loss in glaucoma. [15, 28] Our primary goal in this study was the correlation between IOP and overall RNFL loss. Further analysis incorporating pattern standard deviation (PSD) or visual field index (VFI) could be collected on the study population to assess IOP correlation to visual field changes in treated POAG. This would be beneficial as historical glaucoma clinical trials have used visual fields as the sole end point to determine POAG progression. However, it has been shown that structural damage demonstrable by OCT may often precede detectable associated visual field changes in earlier disease [2, 3]. As we accrue additional longitudinal data, an analysis of visual field data should be possible from follow-up on the study subjects.

The *modified* Goldmann prism holds significant potential for re-establishing IOP and its historic benchmarks as a primary indication of POAG progression. Since both the modified and standard prisms are designed to measure the same IOP in normal healthy eyes with nominal corneas, no reinterpretation of IOP benchmarks is required. Furthermore, any *modified* prism difference in IOP from historic *standard* prism measurements would simply be corrected to account for the original error allowing the practitioner to act accordingly to a new and more accurate IOP. If we consider the diagnosis of NTG as being the false negative of IOP measurement diagnosing glaucoma, a potential demonstration of minimal NTG incidence using the *modified* prism would significantly improve the sensitivity of Goldmann IOP as a primary diagnostic test for glaucoma and a more useful screening tool.

## Data Availability

The deidentified data from the study will be made available upon reasonable request by emailing the corresponding author.

## Abbreviations

IOP: Intraocular Pressure
RNFL: Retinal Nerve Fiber Layer
OCT: Ocular Coherence Tomography
SDOCT: Spectral Domain Ocular Coherence Tomography
CCT: Central Corneal Thickness
POAG: Primary Open Angle Glaucoma
CH: Corneal Hyteresis
GON: Glaucomatous Optic Neuropathy
HVF: Humphrey Visual Field
GAT: Goldmann Applanation Tonometer
EMGT: Early Manifest Glaucoma Trial
OHTS: Ocular Hypertension Treatment Study
AGIS: Advanced Glaucoma Intervention Study
CATS: Correcting Applanation Tonometry Surface
GLME: General Linear Mixed Effects
ModIOP: Modified prism Intraocular Pressure
StdIOP: Standard prism Intraocular Pressure
Mod-StdIOP: Modified Minus Standard prism Differential Intraocular Pressure
EMR: Electronic Medical Records
TSNIT: Temporal Superior Nasal Inferior Temporal
PSD: Pattern Standard Deviation
VFI: Visual Field Index
